# Assessing rates and contextual predictors of 5-year mortality among HIV-infected and HIV-uninfected individuals following HIV testing in Durban, South Africa

**DOI:** 10.1186/s12879-019-4373-9

**Published:** 2019-08-28

**Authors:** Ingrid V. Bassett, Ai Xu, Janet Giddy, Laura M. Bogart, Andrew Boulle, Lucia Millham, Elena Losina, Robert A. Parker

**Affiliations:** 1 Division of Infectious Diseases, Massachusetts General Hospital, 100 Cambridge Street, 16th Floor, Boston, MA 02114 USA; 2 Medical Practice Evaluation Center, Massachusetts General Hospital, Boston, MA USA; 3 Harvard Medical School, Boston, MA USA; 4 Harvard University Center for AIDS Research, Harvard University, Boston, MA USA; 5 Biostatistics Center, Massachusetts General Hospital, Boston, MA USA; 6 McCord Hospital, Durban, South Africa; 7 RAND Corporation, Santa Monica, CA USA; 8 Centre for Infectious Diseases, Epidemiology and Research, School of Public Health and Family Medicine, University of Cape Town, Cape Town, South Africa; 9 Department of Health, Provincial Government of the Western Cape, Cape Town, South Africa; 10 Wellcome Centre for Infectious Diseases Research in Africa, Institute of Infectious Disease and Molecular Medicine, University of Cape Town, Cape Town, South Africa; 11 Department of Orthopedic Surgery, Brigham and Women’s Hospital, Boston, MA USA; 12 Department of Biostatistics, Boston University School of Public Health, Boston, MA USA

**Keywords:** HIV infection, Mortality, Predictors of mortality, Barriers to care

## Abstract

**Background:**

Little is known about contextual factors that predict long-term mortality following HIV testing in resource-limited settings. We evaluated the impact of contextual factors on 5-year mortality among HIV-infected and HIV-uninfected individuals in Durban, South Africa.

**Methods:**

We used data from the Sizanani trial (NCT01188941) in which adults (≥18y) were enrolled prior to HIV testing at 4 outpatient sites. We ascertained vital status via the South African National Population Register. We used random survival forests to identify the most influential predictors of time to death and incorporated these into a Cox model that included age, gender, HIV status, CD4 count, healthcare usage, health facility type, mental health, and self-identified barriers to care (i.e., service delivery, financial, logistical, structural and perceived health).

**Results:**

Among 4816 participants, 39% were HIV-infected. Median age was 31y and 49% were female. 380 of 2508 with survival information (15%) died during median follow-up of 5.8y. For both HIV-infected and HIV-uninfected participants, each additional barrier domain increased the HR of dying by 11% (HR 1.11, 95% CI 1.05–1.18). Every 10-point increase in mental health score decreased the HR by 7% (HR 0.93, 95% CI 0.89–0.97). The hazard ratio (HR) for death of HIV-infected versus HIV-uninfected varied by age: HR of 6.59 (95% CI: 4.79–9.06) at age 20 dropping to a HR of 1.13 (95% CI: 0.86–1.48) at age 60.

**Conclusions:**

Independent of serostatus, more self-identified barrier domains and poorer mental health increased mortality risk. Additionally, the impact of HIV on mortality was most pronounced in younger persons. These factors may be used to identify high-risk individuals requiring intensive follow up, regardless of serostatus.

**Trial registration:**

Clinical Trials.gov Identifier NCT01188941. Registered 26 August 2010.

**Electronic supplementary material:**

The online version of this article (10.1186/s12879-019-4373-9) contains supplementary material, which is available to authorized users.

## Background

South Africa has the largest number of HIV-infected individuals of any country, with over 7 million people diagnosed with HIV and 270,000 new infections in 2016 [1]. People living with HIV (PLWH) who consistently take ART in sub-Saharan Africa can achieve life expectancies similar to those who are HIV-uninfected [2, 3]. However, mortality remains high both before [4, 5] and after ART initiation because of inconsistent care [6–10]. Despite 86% of PLWH in South Africa knowing their HIV status, only 56% were on ART, and only 45% were virally suppressed in 2016 [1]. Thus, accurately ascertaining factors contributing to long-term mortality risk following HIV-diagnosis is paramount.

Studies assessing the long-term risk of mortality in persons living with HIV in sub-Saharan Africa have focused on age, gender, and CD4 counts to evaluate predictors of risk [11–15]. We and others have found, however, that contextual factors (e.g. barriers to care), emotional health, social support, and competing needs at the time of diagnosis, also likely to have an important effect on survival [16, 17]. In addition, most mortality studies, including those that have examined social and contextual factors, have limited analyses to HIV-infected individuals [7, 18–23]. Little is known about contextual factors that predict long-term mortality in resource-limited settings, for both HIV-infected and HIV-uninfected individuals. By comparing to a concurrently enrolled HIV-uninfected group of individuals, who share similar socioeconomic status, we can further our understanding of what interventions in the outpatient setting may improve outcomes in South Africa regardless of HIV status.

Our objective was to assess contextual predictors of 5-year mortality following HIV testing. Research suggests a strong correlation between mental health, especially depression, and mortality among people with HIV [24, 25] and other health conditions [26–28]. Research among PLWH indicates that such associations may be due to a relationship between depression and immune suppression, leading to accelerated disease progression [25, 29, 30]. Moreover, in the US, depression interventions have decreased mortality risk, including for those with chronic diseases, suggesting a causal effect [31–34]. Research also shows strong associations between social support and mortality in general [35, 36]. Thus, we hypothesized that poor emotional health and social support at HIV diagnosis would be associated with higher 5-year mortality rates and could therefore serve as targets for future interventions.

## Methods

### Study setting/design

This analysis includes data from the Sizanani Trial (NCT01188941), a randomized controlled trial that examined the efficacy of health system navigation and short messaging service (SMS) reminders on linkage to and retention in HIV/TB care. We enrolled adults prior to HIV testing at 4 outpatient sites, 2 hospital outpatient departments (one urban and one semi-rural) and 2 primary health clinics (semi-rural) in Durban, South Africa from August 2010–January 2013. This trial is described in detail elsewhere [16, 37, 38]. Because we did not find efficacy of the intervention with respect to linkage to HIV care, TB treatment completion, or 1-year mortality between study arms, we pooled data from the intervention and control groups into a single cohort in the current study and compared them to HIV-uninfected individuals enrolled concurrently.

### Participants

Adults ≥18 years with unknown HIV status presenting for HIV testing were eligible for enrollment. Study enrollment, consisting of informed consent and a baseline questionnaire, occurred prior to HIV testing. This allowed for assessment of contextual factors, emotional health, and social support prior to knowledge of HIV status.

The study was approved by the McCord Hospital Medical Research Ethics Committee, St. Mary’s Hospital Research Ethics Committee, University of KwaZulu- Natal Biomedical Research Ethics Committee, and Partners Institutional Review Board (Protocol 2011-P-001195, Boston, MA).

### Data elements

#### Demographics and CD4 count data

We asked participants to provide demographic information including: age, gender, relationship status, and hours worked outside the home. We collected baseline CD4 count data from medical records for those who were HIV-infected; missing data is discussed in the Additional file 1.

#### Healthcare access

We assessed healthcare access using four questions that determined how difficult it might be for a patient to reach the site. We collected data on mode of transportation (public, private, or other) and distance to clinic. Transportation variables were grouped into two categories – public transport (bus, taxi) or other (including private and other).

#### Healthcare utilization

We assessed self-reported healthcare utilization in the year prior to enrollment, including visits to a community health worker, local clinic, hospital, or private doctor. The total utilization was grouped into three categories: > 5 times, 3–5 times, 1–2 times, did not use healthcare in prior year. We also asked about number of visits to a traditional healer.

#### Health behavior

We asked participants whether they had tested for HIV prior to enrollment. We assessed self-reported competing needs at enrollment, by asking if, in the past 6 months, they had ever gone without healthcare because they needed money for basic needs, or if they had gone without basic needs because they needed money for healthcare [39, 40].

#### Self-perceived barriers to care

We assessed self-perceived barriers to healthcare in the 6 months prior to enrollment using a 12-question instrument modified from the ARTAS-II trial [41]. We grouped barriers into 5 domains: 1) concerns about service delivery (waiting time to see a provider, treatment by clinic staff), 2) financial concerns (ability to afford medication or transportation), 3) perception of personal health (not being sick enough or being too sick), 4) logistical concerns (unable to get out of work, responsibilities to care for others), 5) structural (impaired clinic access due to clinic hours or transportation difficulties, lack of knowledge about where to find care). We created a total number of barriers variable by adding up all barriers in all 5 categories for each participant. We created a similar total number of domains variable by adding up the total number of domains under which a participant indicated they had a barrier.

#### Emotional health and social support

We adapted the 5-item Mental Health Inventory screening test and calculated a mental health composite (MHC) score [42]. In addition, we condensed 13 questions about social support into 4 social support scales (emotional/informational, tangible, positive interaction, and affectionate) and calculated the Social Support Index (SSI) from the Medical Outcomes Study [43]. Separately, we averaged each scale and converted to a scale from 0 to 100. A higher number on the scale indicates better emotional health or social support. An MHC ≤ 52 qualified as a positive depression screen; an SSI below the sample median (75) qualified as a lack of social support [44].

### Outcome ascertainment

We elicited mortality from the National Population Register, which is estimated to incorporate at least 90% of deaths nationwide [13, 45]. We used South African ID numbers (SAIDs) obtained at enrollment to match participants to the National Population Register in November 2017; median follow-up was 5.8 years (IQR 5.2–6.5 years).

### Statistical analysis

We provide a summary of our methods here; further details are in the Additional file 1.

Because a substantial fraction of participants were missing SAIDs, we used propensity score (PS) weighting [46] to make the population with SAIDs be representative of the total group. We estimated the probability of having a SAID from a logistic regression model separately for HIV-infected and HIV-uninfected including all available baseline data, and then used inverse probability weighting to make the population with SAIDs more representative of the total population. To avoid potential confounding of contextual factors by HIV-status, we then used an additional propensity score adjustment so that the HIV-infected and HIV-uninfected were similar. We used random survival forests [47] on all covariates listed in Table 1 to inform the development of our survival model. When the number of variables is comparatively large, a random forest is useful for variable selection because it avoids overfitting [48]. We determined variable importance based on permutation importance and variable depth relative to root node (see Additional file 1).

**Table 1:** Differences between HIV-infected and HIV-uninfected participants at baseline

	Overall, *n* = 4816	HIV-infected, *n* = 1897	HIV-uninfected, *n* = 2919	*P* ^*^
Age, yrs				
Median (IQR)	31.0 (24–41)	33.0 (27–41)	28.0 (22–42)	< 0.001
Sex, n (%)				
Male	2477 [51]	964 (51)	1513 [52]	0.491
Female	2339 [49]	933 (49)	1406 [48]	
Marital status, n (%)				
Never married	3738 (78)	1535 (81)	2203 (76)	< 0.001
Currently married	810 (17)	265 (14)	545 (19)	
Divorce/separated/widowed	239 (5)	85 (5)	154 (5)	
Education, n (%)				
Some high school or greater	4148 (87)	1614 (86)	2534 (87)	0.236
Primary school or less	638 (13)	270 (14)	368 (13)	
Mode of transport, n (%)				
Public transport (bus, taxi)	2283 [48]	877 (47)	1406 [48]	< 0.001
Private transport	1117 [23]	524 (28)	593 (20)	
Other	1387 [29]	484 (26)	903 (31)	
Distance from clinic, n (%)				
Less than 5 km	1177 [25]	352 (19)	825 (28)	< 0.001
At least 5 km	3610 (75)	1533 (81)	2077 (72)	
Health facility type, n (%)				
Primary health clinics	1234 [26]	404 (21)	830 (28)	< 0.001
Hospital outpatient departments	3582 (74)	1493 (79)	2089 (72)	
Work hours outside home, n (%)				
None	2697 [56]	944 (50)	1753 [60]	< 0.001
Less than 40 h	603 (13)	318 (17)	285 (10)	
40 h or more	1516 [32]	635 (34)	881 (30)	
Prior HIV testing, *n* (%)				
Yes	1870 [39]	464 (25)	1406 [48]	< 0.001
No	2917 (61)	1421 (75)	1496 [52]	
Health care use in prior year, *n* (%)				
None	715 (15)	256 (14)	459 (16)	0.006
1–2 times	1499 [31]	570 (30)	929 (32)	
3–5 times	1732 [36]	684 (36)	1048 [36]	
> 5 times	841 (18)	375 (20)	466 (16)	
Visit to traditional healer in prior year, *n* (%)				
Yes	1567 [33]	708 (38)	859 (30)	< 0.001
No	3220 (67)	1177 (62)	2043 (70)	
Social support score				
Median (IQR)	75 (54–87)	67 (50–83)	75 (60–90)	< 0.001
Mental health score				
Median (IQR)	64 (56–80)	64 (56–76)	68 (56–84)	< 0.001
Reported barriers to healthcare, *n* (%)				
Yes	1809 [38]	830 (44)	979 (34)	< 0.001
No	3007 (62)	1067 [56]	1940 (66)	
Number of barriers for participants reporting barriers				
Median (IQR)	3 (2–6)	4 (2–6)	3 (1–5)	< 0.001
Number of barrier domains for participants reporting barriers				
Median (IQR)	3 (1–4)	3 (2–4)	2 (1–4)	< 0.001
Gone without healthcare for basic needs, n (%)				
Yes	920 (19)	414 (22)	506 (17)	< 0.001
No	3896 (81)	1483 (78)	2413 (83)	
Gone without basic needs for healthcare, n (%)				
Yes	724 (15)	323 (17)	401 (14)	0.002
No	4092 (85)	1574 (83)	2518 (86)	

We used a sequential procedure to select the most important variables for the survival model. We first identified the most important of the six domains above to include, and then selected the most important variable(s) in that domain to include in subsequent model construction. Variable selection was based on combining the results from two different statistical approaches. Demographic characteristics were considered the most important category to include, and age was identified as the most important covariate in this category. Gender was selected a priori to be included [49]. Random survival forests were then fitted separately to each of the other five categories of covariates, with age, gender, and HIV status included in all models. The second most important category and important covariates within that category were selected as described above, and the procedure repeated until at least one variable was selected from each category if the category was important. We also evaluated the most important barriers and domains within the self-perceived barriers to care category as detailed in the Additional file 1. Because of the influence of CD4 counts on mortality, we included this in the final model as well. For the 92 HIV-infected participants (8%) missing CD4, we used multiple imputation based on gender, age, health facility type, healthcare use in the past year, total number of domains, and mental health score CD4 counts. HIV-uninfected participants were assigned a CD4 count of 775 based on the median CD4 count of the general population in rural KwaZulu-Natal [50]. We also assessed how the association of health care utilization with mortality varied over the five-year period by fitting the model for the first third, second third, and last third of deaths separately.

We fitted a propensity score-weighted Cox proportional hazards model to the final set of selected covariates. The likelihood ratio test was used to test for interaction effects between HIV status and other covariates included in the Cox model. We used the integrated area under the curve (AUC) as the measure of accuracy for the Cox models [51].

We describe the association of each variable with death using hazard ratios (HR), 95% confidence intervals, and *P*-values in the fully adjusted model (incorporating propensity score weighting and CD4 value imputation), and in simpler models without CD4 value imputation, without propensity score adjustment, and without both to assess the robustness of our conclusions. We used two-tailed *P*-values < 0.05 as a cut-off for statistical significance. Statistical analyses were performed with SAS version 9.4 (SAS Institute, Cary, NC) and “randomForestSRC” in R version 3.4.2 (www.r-project.org) [52].

## Results

### Overall cohort characteristics

There were 4816 enrollees, of whom 1897 (39%) were HIV-infected (Table 1). The median age of the entire cohort was 31 years (IQR: 24–41); 33 years for HIV-infected individuals and 28 years for those HIV-uninfected (*P* < 0.001). Overall, 2339 (49%) were female. The median CD4 count for those with HIV was 196 (IQR: 73–352). Most participants, 3738 (78%), were never married; 2283 (48%) used public transport to travel to the healthcare site and 3582 (74%) underwent HIV testing in an **hospital** outpatient department versus a primary health clinic.

### Self-reported barriers to care

A higher proportion of HIV-infected participants reported one or more barriers to healthcare compared to those HIV-uninfected (44% vs. 34%; *P* < 0.001). Among those who reported any barriers, HIV-infected participants also reported more barriers than HIV-uninfected participants: 4 (IQR: 2–6) vs. 3 (IQR: 1–5; *P* < 0.001). Likewise, for those who reported any barriers, the total number of barriers spanned more domains for HIV-infected participants than for HIV-uninfected individuals: 3 (IQR: 2–4) vs. 2 (IQR: 1–4; *P* < 0.001). 414 (22%) HIV-infected participants had gone without healthcare for money to spend on basic needs (i.e. food, clothing, housing), whereas only 506 (17%) HIV-uninfected participants had done so (*P* < 0.001). Similarly, more HIV-infected individuals 323 (17%) had gone without basic needs for money to spend on healthcare compared to 401 (14%) HIV-uninfected participants (*P* = 0.002).

Patient perception of not being sick enough to seek care (1059; 22%; Table 2) was the most common individual barrier reported. Uniformly, a higher proportion of participants who tested positive for HIV reported experiencing each type of barrier to care than those who tested negative; all differences were statistically significant. The largest discrepancy between HIV-infected and HIV-uninfected individuals was for waiting too long to see a nurse or doctor: 506 (27%) of HIV-infected participants reported this barrier, while only 510 (18%) of HIV-uninfected individuals did (*P* < 0.001).

**Table 2:** Differences in barriers between HIV-infected and HIV-uninfected participants at baseline

	Overall, *n* = 4816	HIV-infected, *n* = 1897	HIV-uninfected, *n* = 2919	*P* ^*^
Barrier domain reported, n (%)				
Service delivery	1152 [24]	566 (30)	586 (20)	< 0.001
Too long to see nurse/MD	1016 [21]	506 (27)	510 (18)	< 0.001
Not treated with respect	179 (4)	93 (5)	86 (3)	0.002
Financial	831 (17)	423 (22)	408 (14)	< 0.001
Cost of transport	731 (15)	377 (20)	354 (12)	< 0.001
Cost of medication	760 (16)	392 (21)	368 (13)	< 0.001
Personal Health	1247 [26]	596 (31)	651 (22)	< 0.001
Not sick enough	1059 [22]	503 (27)	556 (19)	< 0.001
Too sick	433 (9)	244 (13)	189 (7)	< 0.001
Logistical	656 (14)	334 (18)	322 (11)	< 0.001
Could not get off work	382 (8)	213 (11)	169 (6)	< 0.001
Taking care of someone else	371 (8)	182 (10)	189 (7)	< 0.001
Structural	1110 [23]	540 (29)	570 (20)	< 0.001
Did not know where to find care	398 (8)	181 (10)	217 (8)	0.033
Difficult hours	778 (16)	402 (21)	376 (13)	< 0.001
Language barrier	231 (5)	112 (6)	119 (4)	0.014
Transport	688 (14)	353 (19)	335 (12)	< 0.001

The most commonly identified barrier domain was patient perception of personal health, with 1247 (26%) of participants experiencing a barrier in this category. Across barrier domains, service delivery showed the largest difference between HIV-infected and HIV-uninfected participants: 566 (30%) HIV-infected participants identified a service delivery barrier, compared to 586 (20%) HIV-uninfected participants (*P* < 0.001). HIV-infected individuals experienced significantly higher burdens of self-identified barriers across all domains.

### Balance after propensity score adjustment for estimating impact of HIV-infection on mortality

Of 4816 enrollees, only 1154 of HIV-infected (61%) and 1354 of HIV-uninfected (46%) provided valid SAIDs. As shown in Additional file 1: Table S1, there were differences in characteristics between those providing valid SAIDs and those who did not. As shown in Additional file 1: Table S2, initial propensity score weighting of individuals with a valid SAID reduced many of the imbalances between those with a valid SAID and those without valid SAID in both the HIV-infected and HIV-uninfected cohorts. A standard propensity score applied to these weighted population reduced imbalances between HIV-infected and HIV-uninfected participants with valid SAID (Additional file 1: Table S3).

### Predictors of mortality

Regardless of HIV status, patients using primary health clinics, as opposed to those using hospital outpatient departments, had reduced mortality risk (HR: 0.51, 95% CI: 0.38–0.68). A 10-point increase in mental health score decreased the risk of death by 7% (HR: 0.93; 95% CI: 0.89–0.97). Participants who used healthcare services in the year before enrollment in the study were at higher risk of dying, with risk increasing as healthcare use increased (> 5 times, HR: 2.34, 95% CI: 1.75–3.12; 3–5 times, HR: 1.86, 95% CI: 1.42–2.44, 1–2 times, HR: 1.53, 95% CI: 1.15–2.02 compared to no use in the past year). The effect of health care utilization on mortality was reduced over time, showing the most substantial effect in the first third (125 deaths, first 3 months, *P* < 0.001) a smaller but still significant effect in the second third (130 deaths, months 4–21, *P* < 0.001) of deaths and marginal impact in the last third of deaths (125 deaths, month 22 and later, *P* = 0.07).

**Fig. 1:**
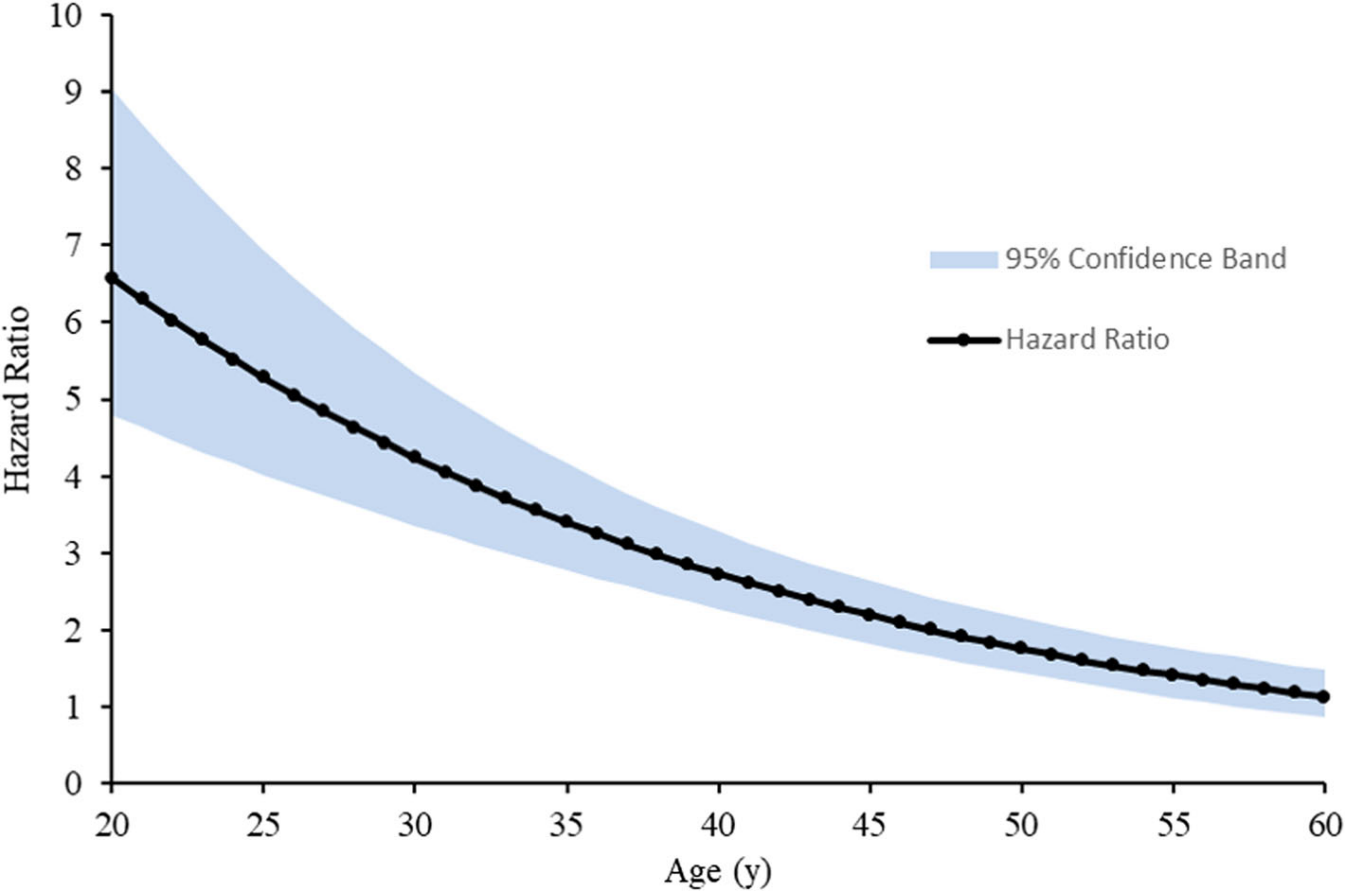
5-year mortality risk among HIV-infected participants varies by age. Hazard ratio is calculated from the primary model, which includes both two-stage propensity score adjustment and multiple imputation of CD4 values in the HIV-infected population

There was a significant interaction of HIV-status with age (HR: 0.63, 95% CI: 0.56–0.70, P < 0.001 for each 10-year increase in age). Each additional 10 years of life increased the risk of death by 94% for HIV-uninfected participants (HR: 1.94, 95% CI: 1.78–2.11), but only by 22% (HR: 1.22, 95% CI: 1.13–1.31; *P* < 0.001) for HIV-infected participants. As shown in Fig. 1, the HR for HIV-infected individuals compared to HIV-uninfected individuals varied from 6.59 (95% CI: 4.79–9.06) at age 20 down to 1.13 (95% CI: 0.86–1.48) at age 60. Sensitivity analyses showed that results were robust to the modeling assumptions with HR for HIV-infection ranging from 6.59 to 7.24 at age 20 and 1.13 to 1.29 at age 60; model details in Table 3.

**Table 3:** Predictors of Mortality Among HIV-infected and HIV-uninfected Patients in Durban, South Africa

Measure	Primary analysis		Alternate analyses		
	PS & CD4 MI HR (95% CI)	*P* ^*ǂ*^	CD4 MI HR (95% CI)	PS HR (95% CI)	Base HR (95% CI)
a. Adjusted hazard ratio of factors in the primary and alternative multivariable models, and in univariate analyses					
HIV Status^x^	**3.86 (3.08–4.85)**	**< 0.001**	3.99 (2.94–5.41)	4.44 (3.51–5.62)	4.54 (3.32–6.21)
Male sex	**1.57 (1.35–1.82)**	**< 0.001**	1.67 (1.35–2.06)	1.67 (1.44–1.93)	1.75 (1.41–2.16)
Test received at primary health clinic	**0.51 (0.38–0.68)**	**< 0.001**	0.60 (0.40–0.91)	0.47 (0.35–0.62)	0.55 (0.37–0.83)
Healthcare use in past year:					
1–2 times	**1.53 (1.15–2.02)**	**0.003**	1.66 (1.09–2.54)	1.65 (1.25–2.18)	1.79 (1.17–2.73)
3–5 times	**1.86 (1.41–2.44)**	**< 0.001**	1.99 (1.32–3.00)	2.01 (1.54–2.63)	2.16 (1.44–3.25)
> 5 times	**2.34 (1.75–3.12)**	**< 0.001**	2.39 (1.55–3.67)	2.63 (1.98–3.49)	2.68 (1.75–4.10)
Total number of healthcare barrier domains reported out of 5 possible	**1.12 (1.07–1.16)**	**< 0.001**	1.11 (1.05–1.18)	1.14 (1.09–1.18)	1.13 (1.06–1.20)
10-point increase in mental health score corresponding to better mental health	**0.93 (0.89–0.97)**	**0.002**	0.94 (0.89–1.01)	0.92 (0.88–0.96)	0.94 (0.88–1.00)
CD4 count (per 100)	**0.80 (0.76–0.84)**	**< 0.001**	0.82 (0.76–0.88)	–	–
b. Adjusted hazard ratios related to the interaction between age and HIV status					
10-year age increase for HIV-infected*	**1.22 (1.13–1.31)**	**< 0.001**	1.20 (1.07–1.35)	1.24 (1.15–1.34)	1.21 (1.08–1.36)
10-year age increase for HIV-uninfected*	**1.94 (1.78–2.11)**	**< 0.001**	1.89 (1.69–2.11)	1.92 (1.77–2.09)	1.87 (1.67–2.09)
HR for HIV-infected at age 20	**6.59 (4.79–9.06)**	**< 0.001**	6.98 (4.43–11.00)	7.11 (5.07–9.96)	7.24 (4.61–11.38)
HR for HIV-infected at age 30	**4.24 (3.35–5.35)**	**< 0.001**	4.45 (3.18–6.22)	4.59 (3.58–5.89)	4.71 (3.38–6.56)
HR for HIV-infected at age 40	**2.72 (2.26–3.28)**	**< 0.001**	2.83 (2.18–3.67)	2.96 (2.46–3.57)	3.06 (2.38–3.94)
HR for HIV-infected at age 50	**1.75 (1.43–2.15)**	**< 0.001**	1.80 (1.38–2.36)	1.91 (1.60–2.29)	1.99 (1.54–2.57)
HR for HIV-infected at age 60	**1.13 (0.86–1.48)**	**< 0.001**	1.15 (0.81–1.63)	1.24 (0.98–1.56)	1.29 (0.92–1.82)

PS: with propensity score adjustment; CD4 MI: with CD4 multiple imputation; HR: adjusted hazard ratio; Base: baseline analysis without propensity score adjustment or CD4 multiple imputation.

^x^ The HIV status variable is calculated as a contrast based on the parameter estimates for the HIV factor,and age factor, incorporating the average difference in age between the HIV+ and HIV- groups. For models including CD4, the HIV status variable also adjusts for the parameter estimate for CD4 and the and the average difference in CD4 between the HIV+ and HIV- groups

^*ǂ*^ *p*-values were generated from Cox proportional hazards models

After adjusting for the HIV x age interaction, there was some evidence for an interaction of HIV-status with number of domains, with the mortality risk for HIV-uninfected individuals increasing by 23% (HR: 1.23, 95% CI: 1.14–1.32) for each additional domain, but more slowly for HIV-infected individuals 7% (HR: 1.07, 95% CI: 1.02–1.13; *P* = 0.002) There was also some evidence that the increased hazard for males was lower for HIV-infected men (HR: 1.38, 95% CI: 1.16–1.65) then for HIV-uninfected men (HR: 2.18, 95% CI: 1.64–2.89; *P* = 0.007). Results for HIV-infected and HIV-uninfected individuals separately are reported in Additional file 1: Table S4.

The integrated AUC for the overall Cox regression model was 0.755. A similar Cox model was fitted separately to the HIV-uninfected and HIV-infected participants with HIV status removed (and CD4 value also removed from the HIV-uninfected population). The model fit was better for HIV-uninfected compared to H IV-infected participants (AUC 0.837 vs. 0.687).

## Discussion

Among 2503 participants with valid SAIDs and complete data enrolled at 4 outpatient sites in Durban, South Africa between 2010 and 2013, more self-identified barrier domains and poorer mental health increase 5-year mortality risk, regardless of HIV status. For every 10-point decrease in mental health score, indicating poorer mental health, 5-year mortality increased by 7%. For each additional self-identified barrier domain 5-year mortality increased by 12%. There was some evidence for an interaction between HIV status and risk of 5-year mortality based on number of reported domains. A higher proportion of HIV-infected participants reported self-identified barriers across every domain when compared to HIV-uninfected participants. Additionally, increased healthcare use in the prior year contributed to increased hazard of death for both HIV-infected and HIV-uninfected individuals. Moreover, those who were HIV-infected had a 4-fold increase in hazard of death during follow-up compared to HIV-uninfected participants at age 31. These results were robust when analyzed across multiple model variations.

This study highlights that barriers to care negatively affect survival regardless of HIV status. HIV-infected individuals reported experiencing significantly more barriers than HIV-uninfected individuals. This could be related to HIV-infected participants having fewer resources as significantly more HIV-infected participants also reported more competing needs than their HIV-uninfected counterparts. Among both HIV-infected and HIV-uninfected individuals, perception of personal health, service delivery, and structural barriers were the most frequently reported barrier domains. Within those domains, both HIV-infected and HIV-uninfected participants most commonly reported waiting too long to see a nurse or doctor or not feeling sick enough to seek care as barriers. Recent studies suggest that improvements in clinic operations, including standardizing staff workloads and patient flow, introducing triage, and increasing staff size might help shorten wait times in low- and middle-income settings [53, 54]. We and others have found that in sub-Saharan African settings, participants often feel as though they are not sick enough to seek care [16, 55] or avoid seeking care when they do not feel ill for fear that treatment will make them feel worse [56]. Efforts to improve clinic operations and to promote seeking routine care may improve long-term mortality in both HIV-infected and HIV-uninfected individuals.

We found that poor mental health also decreased survival regardless of HIV status. Instituting mental health screening not only during HIV testing but also during routine healthcare appointments could allow providers to identify patients with poor mental health and connect them to additional resources. We and others have found that depressive symptoms are common among HIV-infected patients in sub-Saharan Africa and are correlated with decreased likelihoods of obtaining a CD4 count or taking ART [57, 58]. In this study, we used the 5-item Mental Health Inventory screening test; this short survey may be feasible to include in routine healthcare visits. On the other hand, social support was not shown to affect 5-year mortality risk. This may be because we did not measure social support in the form of social integration, which has been shown to be most predictive of mortality [35, 36].

Despite continued efforts to diagnose and link individuals to HIV care in South Africa, HIV-infected individuals remain at substantially increased risk for long-term mortality when compared to their HIV-uninfected counterparts at the same study sites and with similar socioeconomic status. A recent study reported similar findings in Botswana comparing a population-based sample of HIV-infected and -uninfected individuals [59]. Other studies have evaluated long-term mortality risk in HIV-infected individuals in sub-Saharan Africa [11–15], and a few have examined the impact of individual level contextual factors [16, 17]. Unlike previous studies, however, the present study evaluated HIV-infected and HIV-uninfected individuals and found that some factors previously found to be associated with long-term mortality risk in HIV-infected cohorts, are *also* risk factors for HIV-uninfected individuals.

This work should be considered in the context of several limitations. We did not adjust for data on ART use in this model; however, CD4 count was an eligibility criterion for starting ART during the study period and was included in the model. We did not collect data on other health-related comorbidities for either HIV-infected or HIV-uninfected participants; it is possible that those who reported more barriers to care may have also had higher rates of comorbid conditions. We also may underestimate the effects of mental health on mortality because we did not consider psychotic symptoms, which have been associated with an even higher relative risk of mortality compared to anxiety and depression [60]. Healthcare use in the prior year may be on the causal pathway to mortality, however, we felt this variable was an important predictor of time to death. We found that the effect of healthcare use in the prior year on mortality was reduced over time and may be a stronger predictor of early mortality than later mortality. The model results did not change qualitatively when this variable was removed (data not shown). In addition, only 52% of the participants provided a valid SAID number for death registry cross-matching and there were significant differences between those with and without a valid SAID. Though we used propensity scores, we were unable to fully adjust for these differences. Lastly, the methods used to determine predictors of mortality in this study could not be readily used in a clinical setting. In addition to directly addressing those characteristics identified as predictive of mortality, it is necessary to develop predictive instruments, for both HIV-infected and HIV-uninfected individuals, that can be implemented in patient care settings to identify at risk patients.

## Conclusions

HIV infection remains a significant predictor of 5-year mortality in Durban, South Africa. However, additional screening for all patients can be used to help identify at risk individuals who may require additional healthcare interventions. While HIV-infected patients carry a higher burden of self-identified barriers to care than their HIV-uninfected counterparts, the effects of those barriers on mortality risk is not significantly different between HIV-infected and HIV-uninfected individuals. Similarly, while HIV-infected participants reported worse mental health than HIV-uninfected participants, poor mental health increased mortality risk for both groups. Interventions are needed that address both clinic-level barriers to care, such as long wait times, as well as patient-level barriers, including efforts to modify beliefs about the risks of HIV treatment, the benefits of seeking care when feeling healthy, and routine mental health monitoring. Such targeted interventions could improve health outcomes for high-risk individuals. Many structural and logistical barriers can be recognized early, i.e. at the first clinic visit, and could identify patients that may require more intensive follow-up. Both alleviation of barriers that pose increased mortality risks and development of tools to identify high-risk patients in clinical settings could significantly improve outcomes for HIV-infected and HIV-uninfected individuals.

## Additional file

Additional file 1: Statistical analysis and supplementary results tables. Variable selection procedure for random forests. Propensity score adjustment procedure. Imputation of CD4 values. Calculated effect of HIV. Table S1A and 1B – HIV-infected (1A) and HIV-uninfected (1B) comparing those with and without valid SA ID numbers. Table S2A and 2B show the standardized difference between the overall group (HIV-infected or HIV-uninfected) and the group with valid SA ID numbers. (DOCX 74 kb)

## Data Availability

The data supporting these research findings is available upon reasonable request to the corresponding author.

## Abbreviations

AUC: Area under the curve
HR: Hazard ratio
IQR: Interquartile range
MHC: Mental health composite
PLWH: People living with HIV
SAID: South African Identification Number
SMS: Short messaging service
SSI: Social support index
