# Supplementary material for: Assessing rates and contextual predictors of 5-year mortality among HIV-infected and HIV-uninfected individuals following HIV testing in Durban, South Africa
Source: BMC Infect Dis. 2019 Aug 28;19:751. doi: 10.1186/s12879-019-4373-9 (PMC6712739; doi:10.1186/s12879-019-4373-9)
Supplement: Supplementary file 1 — Statistical analysis and supplementary results tables. Variable selection procedure for random forests. Propensity score adjustment procedure. Imputation of CD4 values. Calculated effect of HIV. Table S1A and 1B – HIV-infected (1A) and HIV-uninfected (1B) comparing those with and without valid SA ID numbers. Table S2A and 2B show the standardized difference between the overall group (HIV-infected or HIV-uninfected) and the group with valid SA ID numbers. (DOCX 74 kb) [file 12879_2019_4373_MOESM1_ESM.docx]

**Supplementary Material for the Paper “Assessing rates and contextual predictors of 5-year mortality among HIV-infected and HIV-uninfected individuals following HIV testing in Durban, South Africa”**

In the following, we provide additional results to supplement our main submission.

**Statistical Analysis.**

*Variable selection procedure for random forests*

Random forests estimate the importance of predictors using a non-parametric approach and have fewer assumptions.^1^ We based variable selection on two different measures of variable importance within a category. Permutation importance (VIMP) is the difference between out-of-box (OOB; cases not used to develop the specific model) prediction error before and after variable permutation.^1^ Variables with larger permutation importance have more predictive ability. Minimal depth of a maximal subtree,^2^ another measure of variable importance, measures the average depth of a variable relative to the root of the tree. Variables that split closer to the root node (lower minimal depth) are more important predictors. We separately ranked the VIMP and minimal depth from most to least important and used the sum of the two ranks for variable selection.

*Propensity score adjustment procedure*

We used a two-step procedure using propensity scores, so that the HIV+ and HIV- populations being compared were as similar as possible to minimize potential residual confounding factors to estimate predictors of mortality. As shown in Table 1 of the paper, the HIV+ and HIV- populations differed on almost all characteristics. Furthermore, as shown separately for the HIV+ and HIV- populations (Supplementary Table 1A and 1B, respectively) those with valid SA ID numbers were different on virtually all characteristics from those who declined to provide this information. Thus, our first step was to make the population with a valid SA ID number as representative as possible of the entire group (HIV+ or HIV-) in the original study. We calculated the probability that an individual provided a valid SA ID number using a logistic regression including all variables listed in Table 1, adding presence and value of CD4 for the HIV-infected population. Unlike a traditional propensity score approach, in which all observations are then weighted, we applied the weights only to individuals with a valid SA ID number. The initial weight used was 1/probability of having a valid SA ID number, with truncation at 20 for observations with a very low probability of having a valid SA ID number. We then multiplied the weight with a constant multiplier so that the sum of the weights equaled the number of individuals in the total population.

Supplementary Tables 2A and 2B show the standardized difference between the overall group (HIV-infected or HIV-uninfected) and the group with valid SA ID numbers. The standardized difference is the absolute value of the difference between the total group (HIV-infected or -uninfected) and either the original or weighted group divided by the standard deviation of this estimate; a smaller number is better. The tables show that this procedure substantially reduced, but did not eliminate, the difference between those with a valid SA ID number and the total group. In summary, for the HIV-infected population, the standardized difference for the unweighted group with a valid SA ID number ranged from 0.001-8.964, median 1.134, which was reduced to 0.001-2.040, median 0.179 after weighting. Similarly, for the HIV-uninfected population the standardized difference was reduced from 0.032-11.378, median 2.577 to 0.002-2.506, median 0.442 by weighting.

In the second step, we used a standard propensity score approach to make the weighted populations with valid SA ID numbers as similar as possible between the HIV+ and HIV- groups on the covariates included in Table 1. This was intended to minimize residual confounding from HIV-status when estimating the impact of the various contextual factors. In this step, using a weighted logistic regression analysis on the pooled population of individuals with a valid SA ID number, we estimated the probability that an individual was HIV+ and then applied the standard propensity score inverse probability weighting to individuals in both groups.

For clarity we also describe this in mathematical notation. Let S stand for + for HIV positive and - for HIV negative respectively, let N_s_ be the total number of individuals in the original study with HIV status S, and *iS* indicate person *iS* = 1, … , N_s_. In step 1, we calculate

e_1iS_ = Probability(valid SA ID number | S)

for each individual *iS* using a separate logistic regression for each status S. The initial weight for an individual is then

w_1iS_ = min(20, 1/e_1iS_) if person *iS* has a valid SA ID number

w_1iS_ = 0 otherwise

and finally a constant multiplier of

Ns / Σ w_1iS_

is then applied to all weights so that at the end of this step the subgroup with valid SA ID numbers is weighted to represent the total initial population and be of the same size.

In step 2, using a logistic regression for the population with valid SA ID numbers only, using the weighting in step 1, we calculated

e_2iS_ = Probability(HIV Positive)

and then use standard inverse probability of HIV+ weighting for those with valid SA ID numbers:

w_2i+_ = min(20, 1/e_2i+_) for HIV+ individuals

and

w_2i-_ = min(20, 1/(1-e_2i-_) for HIV- individuals.

*Imputation of CD4 values*

In the analysis, 92 out of 1154 (8%) of the HIV-infected participants were missing CD4 data. Multiple imputation^3^ was used based on selected variables (gender, age, health facility type, healthcare use in past year, total number of domains, mental health score) through MCMC method with five imputations. HIV-uninfected participants were assigned a CD4 count of 775 based on the median CD4 count of the general population in rural KwaZulu-Natal.^4^

*Calculated effect of HIV*

Because the difference in CD4 levels and age between HIV-infected and HIV-uninfected individuals was associated with the estimated HIV effect in the full model, it was necessary to calculate an adjusted effect of HIV from the Cox model. This was done using a contrast in SAS® combining the coefficient for the effect of CD4 and age multiplied by the structural difference between the two groups with the estimated effect of HIV in the model with CD4 and age and is calculated from the following formula:.

$$\hat{\beta}_{Adjusted HIV}=\hat{\beta}_{HIV}+\left( \bar{CD4}_{HIV+}-\bar{CD4}_{HIV-} \right)\times\hat{\beta}_{CD4}+\left( \bar{Age}_{HIV+}-\bar{Age}_{HIV-} \right)\times\hat{\beta}_{Age}$$

where $\hat{\beta}_{HIV}$is the effect estimate of the HIV term in the model and $\bar{CD4}_{HIV+}$is the average value of CD4 in the HIV+ population, etc. The standard error of this contrast appropriately accounts for the correlation within the three parameter estimates as well as the variability of each estimate.

**References**

1. Breiman L. Random Forests. *Mach Learn.* 2001;45(1):5-32.

2. Ishwaran H, Kogalur U, Gorodeski E, Minn A, Lauer M. High-Dimensional Variable Selevtion for Survival Data. *J Am Stat Assoc.* 2010(105):205-217.

3. Rubin DB. Multiple Imputation After 18+ Years. *J Am Stat Assoc.* 1996;91(434):473-489.

4. Malaza A, Mossong J, Bärnighausen T, Viljoen J, Newell M-L. Population-Based CD4 Counts in a Rural Area in South Africa with High HIV Prevalence and High Antiretroviral Treatment Coverage. *PloS One.* 2013;8(7):e70126.

| **Table S1a.** Differences between participants with/without valid South African ID numbers (HIV-infected participants) at baseline | | | | |
| --- | --- | --- | --- | --- |
|  | Overall, n=1897 | Valid SAID, n=1154 | Non-valid SAID, n=743 | *P* |
| Age, yrs |  |  |  |  |
| Median (IQR) | 33 (27-41) | 35 (28-42) | 31 (26-39) | <0.001 |
| Sex, n (%) |  |  |  |  |
| Male | 964 (51) | 605 (52) | 359 (48) | 0.081 |
| Female | 933 (49) | 549 (48) | 384 (52) |  |
| Marital status, n (%) |  |  |  |  |
| Never married | 1535 (81) | 897 (78) | 638 (87) | <0.001 |
| Currently married | 265 (14) | 190 (17) | 75 (10) |  |
| Divorce/separated/  widowed | 85 (5) | 62 (5) | 23 (3) |  |
| Education, n (%) |  |  |  |  |
| Some high school or  greater | 1614 (86) | 1014 (88) | 600 (82) | <0.001 |
| Primary school or less | 270 (14) | 134 (12) | 136 (19) |  |
| Mode of transport, n (%) |  |  |  |  |
| Public transport (bus,  taxi) | 877 (47) | 536 (47) | 341 (46) | <0.001 |
| Private transport | 524 (28) | 415 (36) | 109 (15) |  |
| Other | 484 (26) | 198 (17) | 286 (39) |  |
| Distance from clinic, n (%) |  |  |  |  |
| Less than 5 km | 352 (19) | 169 (15) | 183 (25) | <0.001 |
| At least 5 km | 1533 (81) | 980 (85) | 553 (75) |  |
| Health facility type, n (%) |  |  |  |  |
| Primary health clinics | 404 (21) | 160 (14) | 244 (33) | <0.001 |
| Outpatient departments | 1493 (79) | 994 (86) | 499 (67) |  |
| Work hours outside home, n (%) |  |  |  |  |
| None | 944 (50) | 455 (39) | 489 (66) | <0.001 |
| Less than 40 hours | 318 (17) | 239 (21) | 79 (11) |  |
| 40 hours or more | 635 (34) | 460 (40) | 175 (24) |  |
| Prior HIV testing, n (%) |  |  |  |  |
| Yes | 464 (25) | 265 (23) | 199 (27) | 0.059 |
| No | 1421 (75) | 884 (77) | 537 (73) |  |
| Health care use in prior year, n (%) |  |  |  |  |
| None | 256 (14) | 181 (16) | 75 (10) | <0.001 |
| 1-2 times | 570 (30) | 312 (27) | 258 (35) |  |
| 3-5 times | 684 (36) | 417 (36) | 267 (36) |  |
| >5 times | 375 (20) | 239 (21) | 136 (19) |  |
| Visit to traditional healer in prior year, n (%) |  |  |  |  |
| Yes | 708 (38) | 422 (37) | 286 (39) | 0.255 |
| No | 1177 (62) | 727 (63) | 450 (61) |  |
| Social support score |  |  |  |  |
| Median (IQR) | 67 (50-83) | 64 (50-81) | 75 (52-85) | 0.030 |
| Mental health score |  |  |  |  |
| Median (IQR) | 64 (56-76) | 64 (56-76) | 64 (56-76) | 0.831 |
| Reported barriers to healthcare, n (%) |  |  |  |  |
| Yes | 830 (44) | 444 (38) | 386 (52) | <0.001 |
| No | 1067 (56) | 710 (62) | 357 (48) |  |
| Number of barriers for participants reporting barriers |  |  |  |  |
| Median (IQR) | 4 (2-6) | 4 (2-5) | 4 (2-7) | <0.001 |
| Number of barrier domains for participants reporting barriers |  |  |  |  |
| Median (IQR) | 3 (2-4) | 3 (2-4) | 3 (2-4) | 0.008 |
| Gone without healthcare for basic needs, n (%) |  |  |  |  |
| Yes | 414 (22) | 224 (19) | 190 (26) | 0.002 |
| No | 1483 (78) | 930 (81) | 553 (74) |  |
| Gone without basic needs for healthcare, n (%) |  |  |  |  |
| Yes | 323 (17) | 183 (16) | 140 (19) | 0.091 |
| No | 1574 (83) | 971 (84) | 603 (81) |  |
| CD4 Results* | 244 (216) | 243 (223) | 245 (205) | 0.850 |

*Missing 194 out of 1897 (92 missing in valid group, 102 missing in non-valid group)

| **Table S1b.** Differences between participants with/without valid South African ID numbers (HIV-uninfected participants) at baseline | | | | |
| --- | --- | --- | --- | --- |
|  | Overall, n=2919 | Valid SAID, n=1354 | Non-valid SAID, n=1565 | *P* |
| Age, yrs |  |  |  |  |
| Median (IQR) | 28 (22-42) | 30 (23-44) | 27 (22-39) | <0.001 |
| Sex, n (%) |  |  |  |  |
| Male | 1513 (52) | 728 (54) | 785 (50) | 0.052 |
| Female | 1406 (48) | 626 (46) | 780 (50) |  |
| Marital status, n (%) |  |  |  |  |
| Never married | 2203 (76) | 983 (73) | 1220 (79) | <0.001 |
| Currently married | 545 (19) | 279 (21) | 266 (17) |  |
| Divorce/separated/  widowed | 154 (5) | 92 (7) | 62 (4) |  |
| Education, n (%) |  |  |  |  |
| Some high school or  greater | 2534 (87) | 1217 (90) | 1317 (85) | <0.001 |
| Primary school or less | 368 (13) | 137 (10) | 231 (15) |  |
| Mode of transport, n (%) |  |  |  |  |
| Public transport (bus,  taxi) | 1406 (48) | 612 (45) | 794 (51) | <0.001 |
| Private transport | 593 (20) | 391 (29) | 202 (13) |  |
| Other | 903 (31) | 351 (26) | 552 (36) |  |
| Distance from clinic, n (%) |  |  |  |  |
| Less than 5 km | 825 (28) | 342 (25) | 483 (31) | <0.001 |
| At least 5 km | 2077 (72) | 1012 (75) | 1065 (69) |  |
| Health facility type, n (%) |  |  |  |  |
| Primary health clinics | 830 (28) | 292 (22) | 538 (34) | <0.001 |
| Outpatient departments | 2089 (72) | 1062 (78) | 1027 (66) |  |
| Work hours outside home, n (%) |  |  |  |  |
| None | 1753 (60) | 672 (50) | 1081 (69) | <0.001 |
| Less than 40 hours | 285 (10) | 155 (11) | 130 (8) |  |
| 40 hours or more | 881 (30) | 527 (39) | 354 (23) |  |
| Prior HIV testing, n (%) |  |  |  |  |
| Yes | 1406 (48) | 742 (55) | 664 (43) | <0.001 |
| No | 1496 (52) | 612 (45) | 884 (57) |  |
| Health care use in prior year, n (%) |  |  |  |  |
| None | 459 (16) | 264 (20) | 195 (13) | <0.001 |
| 1-2 times | 929 (32) | 373 (28) | 556 (36) |  |
| 3-5 times | 1048 (36) | 476 (35) | 572 (37) |  |
| >5 times | 466 (16) | 241 (18) | 225 (15) |  |
| Visit to traditional healer in prior year, n (%) |  |  |  |  |
| Yes | 859 (30) | 339 (25) | 520 (34) | <0.001 |
| No | 2043 (70) | 1015 (75) | 1028 (66) |  |
|  |  |  |  |  |
| Social support score |  |  |  |  |
| Median (IQR) | 75 (60-90) | 77 (64-96) | 75 (58-89) | <0.001 |
| Mental health score |  |  |  |  |
| Median (IQR) | 68 (56-84) | 72 (60-88) | 64 (56-80) | <0.001 |
| Reported barriers to healthcare, n (%) |  |  |  |  |
| Yes | 979 (34) | 392 (29) | 587 (38) | <0.001 |
| No | 1940 (66) | 962 (71) | 978 (62) |  |
| Number of barriers for participants reporting barriers |  |  |  |  |
| Median (IQR) | 3 (1-5) | 3 (1-5) | 3 (2-5) | 0.126 |
| Number of barrier domains for participants reporting barriers |  |  |  |  |
| Median (IQR) | 2 (1-4) | 2 (1-4) | 2 (1-4) | 0.218 |
| Gone without healthcare for basic needs, n (%) |  |  |  |  |
| Yes | 506 (17) | 201 (15) | 305 (20) | <0.001 |
| No | 2413 (83) | 1153 (85) | 1260 (81) |  |
| Gone without basic needs for healthcare, n (%) |  |  |  |  |
| Yes | 401 (14) | 163 (12) | 238 (15) | 0.013 |
| No | 2518 (86) | 1191 (88) | 1327 (85) |  |

| **Table S2a.** Impact of initial weighting on representativeness of those with valid South African ID numbers (HIV-infected participants) | | | | | |
| --- | --- | --- | --- | --- | --- |
|  | Overall, n=1897 | After weighting | Unweighted group | Standardized difference after weighting | Standardized difference for unweighted group |
| Age, yrs |  |  |  |  |  |
| Mean (SD) | 35 (10) | 35 (13) | 36 (10) | -0.00 | -0.07 |
| Sex, (%) |  |  |  |  |  |
| Female | 49 | 50 | 48 | -0.32 | 0.86 |
| Marital status, (%) |  |  |  |  |  |
| Never married | 81 | 81 | 78 | 0.29 | 2.22 |
| Currently married | 14 | 14 | 17 | -0.21 | -1.83 |
| Divorced/separated/  widowed | 5 | 5 | 5 | -0.18 | -1.08 |
| Education, (%) |  |  |  |  |  |
| Some high school  or greater | 86 | 86 | 88 | -0.17 | -2.14 |
| Mode of transport, (%) |  |  |  |  |  |
| Public transport | 47 | 45 | 47 | 0.62 | -0.07 |
| Private transport | 28 | 29 | 36 | -0.87 | -4.75 |
| Other | 26 | 25 | 17 | 0.20 | 5.62 |
| Distance from clinic, (%) |  |  |  |  |  |
| Less than 5 km | 19 | 18 | 15 | 0.49 | 2.88 |
| Health facility type, (%) |  |  |  |  |  |
| Primary health clinics | 21 | 21 | 14 | 0.54 | 5.37 |
| Work hours outside home, (%) |  |  |  |  |  |
| None | 50 | 50 | 39 | 0.02 | 5.62 |
| Less than 40 hours | 17 | 18 | 21 | -0.67 | -2.69 |
| 40 hours or more | 33 | 33 | 40 | 0.52 | -3.54 |
| Prior HIV testing, (%) |  |  |  |  |  |
| Yes | 25 | 24 | 23 | 0.29 | 0.98 |
| Health care use in prior year, (%) |  |  |  |  |  |
| None | 14 | 14 | 16 | -0.00 | -1.63 |
| 1-2 times | 30 | 29 | 27 | 0.60 | 1.83 |
| 3-5 times | 36 | 36 | 36 | -0.02 | -0.00 |
| >5 times | 20 | 21 | 21 | -0.66 | -0.60 |
| Visit to traditional healer in prior year, (%) |  |  |  |  |  |
| Yes | 38 | 37 | 37 | 0.07 | 0.46 |
| Social support score |  |  |  |  |  |
| Mean (SD) | 66 (22) | 67 (28) | 65 (21) | -0.02 | 0.03 |
| Mental health score |  |  |  |  |  |
| Mean (SD) | 66 (16) | 66 (20) | 66 (15) | -0.01 | -0.00 |
| Reported barriers to healthcare, (%) |  |  |  |  |  |
| Yes | 44 | 42 | 38 | 0.87 | 2.88 |
| Number of barriers for participants reporting barriers |  |  |  |  |  |
| Mean (SD) | 4 (3) | 4 (4) | 4 (2) | -0.04 | 0.09 |
| Number of barrier domains for participants reporting barriers |  |  |  |  |  |
| Mean (SD) | 3 (1) | 3 (2) | 3 (1) | -0.05 | 0.06 |
| Gone without healthcare for basic needs, (%) |  |  |  |  |  |
| Yes | 22 | 22 | 19 | -0.26 | 1.61 |
| Gone without basic needs for healthcare, (%) |  |  |  |  |  |
| Yes | 17 | 17 | 16 | -0.11 | 0.85 |
| Service delivery, (%) |  |  |  |  |  |
| Yes | 30 | 30 | 26 | -0.05 | 2.47 |
| Financial, (%) |  |  |  |  |  |
| Yes | 22 | 23 | 19 | -0.70 | 2.34 |
| Personal health, (%) |  |  |  |  |  |
| Yes | 31 | 31 | 27 | 0.03 | 2.54 |
| Logistical, (%) |  |  |  |  |  |
| Yes | 18 | 18 | 15 | -0.47 | 2.11 |
| Structural, (%) |  |  |  |  |  |
| Yes | 28 | 28 | 23 | -0.04 | 3.35 |
| CD4 count |  |  |  |  |  |
| Mean (SD) | 239 (206) | 239 (268) | 239 (215) | 0.00 | -0.00 |

| **Table S2b.** Impact of initial weighting on representativeness of those with valid South African ID numbers (HIV-uninfected participants) | | | | | |
| --- | --- | --- | --- | --- | --- |
|  | Overall, n=2919 | After weighting | Unweighted group | Standardized difference after weighting | Standardized difference for unweighted group |
| Age, yrs |  |  |  |  |  |
| Mean (SD) | 33 (14) | 33 (20) | 34 (14) | -0.00 | -0.07 |
| Sex, (%) |  |  |  |  |  |
| Female | 48 | 48 | 52 | 0.35 | -1.71 |
| Marital status, (%) |  |  |  |  |  |
| Never married | 76 | 76 | 87 | -0.21 | -7.26 |
| Currently married | 19 | 19 | 10 | -0.18 | 6.46 |
| Divorced/separated/  widowed | 5 | 5 | 3 | 0.71 | 2.85 |
| Education, (%) |  |  |  |  |  |
| Some high school  or greater | 87 | 87 | 82 | 0.24 | 3.72 |
| Mode of transport, (%) |  |  |  |  |  |
| Public transport | 48 | 47 | 46 | 0.80 | 1.03 |
| Private transport | 20 | 20 | 15 | 0.58 | 3.73 |
| Other | 31 | 33 | 39 | -1.36 | -3.89 |
| Distance from clinic, (%) |  |  |  |  |  |
| Less than 5 km | 28 | 29 | 25 | -0.18 | 1.98 |
| Health facility type, (%) |  |  |  |  |  |
| Primary health clinics | 28 | 30 | 33 | -1.19 | -2.30 |
| Work hours outside home, (%) |  |  |  |  |  |
| None | 60 | 62 | 66 | -1.36 | -2.94 |
| Less than 40 hours | 10 | 9 | 11 | 0.58 | -0.69 |
| 40 hours or more | 30 | 29 | 24 | 1.08 | 3.74 |
| Prior HIV testing, (%) |  |  |  |  |  |
| Yes | 48 | 49 | 27 | -0.15 | 11.38 |
| Health care use in prior year, (%) |  |  |  |  |  |
| None | 16 | 15 | 10 | 0.49 | 4.31 |
| 1-2 times | 32 | 32 | 35 | 0.38 | -1.55 |
| 3-5 times | 36 | 37 | 36 | -0.46 | -0.08 |
| >5 times | 16 | 16 | 18 | -0.36 | -1.53 |
| Visit to traditional healer in prior year, (%) |  |  |  |  |  |
| Yes | 30 | 29 | 39 | 0.42 | -4.66 |
| Social support score |  |  |  |  |  |
| Mean (SD) | 74 (21) | 74 (31) | 76 (20) | -0.01 | -0.08 |
| Mental health score |  |  |  |  |  |
| Mean (SD) | 70 (18) | 70 (27) | 72 (18) | -0.01 | -0.09 |
| Reported barriers to healthcare, (%) |  |  |  |  |  |
| Yes | 34 | 34 | 52 | -0.53 | -9.07 |
| Number of barriers for participants reporting barriers |  |  |  |  |  |
| Mean (SD) | 4 (2) | 4 (4) | 3 (2) | -0.05 | 0.03 |
| Number of barrier domains for participants reporting barriers |  |  |  |  |  |
| Mean (SD) | 3 (1) | 3 (2) | 3 (1) | -0.05 | 0.03 |
| Gone without healthcare for basic needs, (%) |  |  |  |  |  |
| Yes | 17 | 20 | 26 | -2.15 | -4.71 |
| Gone without basic needs for healthcare, (%) |  |  |  |  |  |
| Yes | 14 | 15 | 19 | -1.76 | -3.25 |
| Service delivery, (%) |  |  |  |  |  |
| Yes | 20 | 22 | 36 | -1.42 | -8.43 |
| Financial, (%) |  |  |  |  |  |
| Yes | 14 | 17 | 28 | -2.51 | -7.80 |
| Personal health, (%) |  |  |  |  |  |
| Yes | 22 | 23 | 38 | -0.92 | -8.13 |
| Logistical, (%) |  |  |  |  |  |
| Yes | 11 | 12 | 22 | -1.52 | -6.78 |
| Structural, (%) |  |  |  |  |  |
| Yes | 20 | 22 | 37 | -1.79 | -9.06 |

| **Table S3a.** Comparison of HIV-infected and HIV-uninfected participants with valid South African ID number using initial weights | | | |
| --- | --- | --- | --- |
|  | Weighted HIV-infected with valid SAID | Weighted HIV-uninfected with valid SAID | Standardized difference between weighted HIV-infected and HIV-uninfected |
| Age, yrs |  |  |  |
| Mean (SD) | 33 (20) | 35 (13) | -0.08 |
| Sex, (%) |  |  |  |
| Female | 48 | 50 | -1.20 |
| Marital status, (%) |  |  |  |
| Never married | 76 | 81 | -3.70 |
| Currently married | 19 | 14 | 4.08 |
| Divorced/separated/  widowed | 5 | 5 | 0.00 |
| Education, (%) |  |  |  |
| Some high school  or less | 87 | 86 | 0.88 |
| Mode of transport, (%) |  |  |  |
| Public transport | 47 | 45 | 1.20 |
| Private transport | 20 | 29 | -6.24 |
| Other | 33 | 25 | 5.34 |
| Distance from clinic, (%) |  |  |  |
| Less than 5 km | 29 | 18 | 8.00 |
| Health facility type, (%) |  |  |  |
| Primary health clinics | 30 | 21 | 6.34 |
| Work hours outside home, (%) |  |  |  |
| None | 62 | 50 | 7.30 |
| Less than 40 hours | 9 | 18 | -7.79 |
| 40 hours or more | 29 | 33 | -2.60 |
| Prior HIV testing, (%) |  |  |  |
| Yes | 49 | 24 | 16.38 |
| Health care use in prior year, (%) |  |  |  |
| None | 15 | 14 | 0.86 |
| 1-2 times | 32 | 29 | 1.96 |
| 3-5 times | 37 | 36 | 0.62 |
| >5 times | 16 | 21 | -3.83 |
| Visit to traditional healer in prior year, (%) |  |  |  |
| Yes | 29 | 37 | -5.09 |
| Social support score |  |  |  |
| Mean (SD) | 74 (31) | 67 (28) | 0.18 |
| Mental health score |  |  |  |
| Mean (SD) | 70 (27) | 66 (20) | 0.12 |
| Reported barriers to healthcare, (%) |  |  |  |
| Yes | 34 | 42 | -4.95 |
| Number of barriers for participants reporting barriers |  |  |  |
| Mean (SD) | 4 (4) | 4 (4) | -0.12 |
| Number of barrier domains for participants reporting barriers |  |  |  |
| Mean (SD) | 3 (2) | 3 (2) | -0.12 |
| Gone without healthcare for basic needs, (%) |  |  |  |
| Yes | 20 | 22 | -1.47 |
| Gone without basic needs for healthcare, (%) |  |  |  |
| Yes | 15 | 17 | -1.62 |
| Service delivery, (%) |  |  |  |
| Yes | 22 | 30 | -5.46 |
| Financial, (%) |  |  |  |
| Yes | 17 | 23 | -4.48 |
| Personal health, (%) |  |  |  |
| Yes | 23 | 31 | -5.37 |
| Logistical, (%) |  |  |  |
| Yes | 12 | 18 | -4.95 |
| Structural, (%) |  |  |  |
| Yes | 22 | 29 | -4.83 |

| **Table S3b.** Comparison of HIV-infected and HIV-uninfected participants with valid South African ID number using second stage weights | | | |
| --- | --- | --- | --- |
|  | Weighted HIV-infected with valid SAID | Weighted HIV-uninfected with valid SAID | Standardized difference between weighted HIV-infected and HIV-uninfected |
| Age, yrs |  |  |  |
| Mean (SD) | 35 (18) | 35 (16) | 0.01 |
| Sex, (%) |  |  |  |
| Female | 46 | 46 | 0.00 |
| Marital status, (%) |  |  |  |
| Never married | 75 | 70 | 3.48 |
| Currently married | 19 | 24 | -3.79 |
| Divorced/separated/  widowed | 6 | 6 | 0.00 |
| Education, (%) |  |  |  |
| Some high school  or greater | 89 | 89 | 0.00 |
| Mode of transport, (%) |  |  |  |
| Public transport | 45 | 50 | -3.10 |
| Private transport | 31 | 29 | 1.34 |
| Other | 24 | 22 | 1.47 |
| Distance from clinic, (%) |  |  |  |
| Less than 5 km | 23 | 22 | 0.74 |
| Health facility type, (%) |  |  |  |
| Primary health clinics | 19 | 18 | 0.80 |
| Work hours outside home, (%) |  |  |  |
| None | 45 | 47 | -1.24 |
| Less than 40 hours | 16 | 15 | 0.85 |
| 40 hours or more | 39 | 38 | 0.63 |
| Prior HIV testing, (%) |  |  |  |
| Yes | 45 | 44 | 0.62 |
| Health care use in prior year, (%) |  |  |  |
| None | 19 | 19 | 0.00 |
| 1-2 times | 27 | 29 | -1.38 |
| 3-5 times | 35 | 35 | 0.00 |
| >5 times | 19 | 18 | 0.80 |
| Visit to traditional healer in prior year, (%) |  |  |  |
| Yes | 28 | 29 | -0.68 |
| Social support score |  |  |  |
| Mean (SD) | 73 (26) | 71 (34) | 0.06 |
| Mental health score |  |  |  |
| Mean (SD) | 70 (22) | 69 (26) | 0.04 |
| Reported barriers to healthcare, (%) |  |  |  |
| Yes | 32 | 32 | 0.00 |
| Number of barriers for participants reporting barriers |  |  |  |
| Mean (SD) | 4 (4) | 4 (3) | 0.01 |
| Number of barrier domains for participants reporting barriers |  |  |  |
| Mean (SD) | 3 (2) | 3 (2) | 0.00 |
| Gone without healthcare for basic needs, (%) |  |  |  |
| Yes | 16 | 17 | -0.84 |
| Gone without basic needs for healthcare, (%) |  |  |  |
| Yes | 13 | 13 | 0.00 |
| Service delivery, (%) |  |  |  |
| Yes | 19 | 19 | 0.00 |
| Financial, (%) |  |  |  |
| Yes | 14 | 14 | 0.00 |
| Personal health, (%) |  |  |  |
| Yes | 23 | 23 | 0.00 |
| Logistical, (%) |  |  |  |
| Yes | 11 | 10 | 1.01 |
| Structural, (%) |  |  |  |
| Yes | 20 | 19 | 0.78 |

| **Table S4.** Predictors of Mortality Among HIV-infected and HIV-uninfected Patients in Durban, South Africa using separate models for HIV-infected and HIV-uninfected groups | | | | |
| --- | --- | --- | --- | --- |
|  | **HIV-infected** | | **HIV-uninfected** | |
| **Measure** | **HR (95% CI)** | ***P ^ǂ^*** | **HR (95% CI)** | ***P ^ǂ^*** |
| 10-year age increase | 1.23 (1.12-1.34) | <0.001 | 1.82 (1.67-1.98) | <0.001 |
| Male sex | 1.23 (1.01-1.50) | 0.038 | 2.08 (1.59-2.73) | <0.001 |
| Test received at primary health clinic | 0.58 (0.42-0.82) | 0.002 | 0.47 (0.29-0.77) | 0.002 |
| Healthcare use in past year: |  |  |  |  |
| 1-2 times | 1.61 (1.06-2.44) | 0.026 | 0.97 (0.55-1.73) | 0.930 |
| 3-5 times | 1.90 (1.26-2.85) | 0.002 | 1.86 (1.08-3.21) | 0.025 |
| >5 times | 2.26 (1.47-3.47) | <0.001 | 2.70 (1.53-4.77) | <0.001 |
| Total number of healthcare barrier domains reported out of 5 possible | 1.08 (1.02-1.14) | 0.009 | 1.17 (1.09-1.25) | <0.001 |
| 10-point increase in mental health score corresponding to better mental health | 0.98 (0.92-1.04) | 0.471 | 0.97 (0.91-1.05) | 0.483 |
| CD4 count (per 100) | 0.78 (0.73-0.84) | <0.001 | -- | -- |
